# Corrigendum to: A novel fully automated MRI-based deep-learning method for classification of 1p/19q co-deletion status in brain gliomas

**DOI:** 10.1093/noajnl/vdac187

**Published:** 2023-01-10

**Authors:** Chandan Ganesh Bangalore Yogananda, Bhavya R Shah, Frank F Yu, Marco C Pinho, Sahil S Nalawade, Gowtham K Murugesan, Benjamin C Wagner, Bruce Mickey, Toral R Patel, Baowei Fei, Ananth J Madhuranthakam, Joseph A Maldjian

This is a correction to: Neuro-Oncology Advances, Volume 2, Issue Supplement 4, December 2020, doi: 10.1093/noajnl/vdaa066.

There was an error in the python code for the 3-fold cross validation procedure. This resulted in the use of the training cases instead of the set-aside test cases for the molecular marker accuracy testing procedure. This caused our reported accuracies from the TCIA/TCGA data set to be artificially inflated. The corrected accuracies for Table 1 (computed using nnU-Net^1^), along with the updated ROC curve for Figure 3 are provided here. The updated accuracies, while encouraging, do not outperform other reported methods for 1p/19q molecular marker prediction using MRI.

**Table 1:** Cross-validation results

Fold Description	1p/19q-net		
Fold Number	%Accuracy	AUC	Dice Score
Fold 1	78.68	0.7525	0.8472
Fold 2	79.83	0.7461	0.8551
Fold 3	84.42	0.8503	0.8333
**Average**	**80.97 ± 0.03**	**0.78 ± 0.058**	**0.8452 ± 0.011**

**Figure 3.**
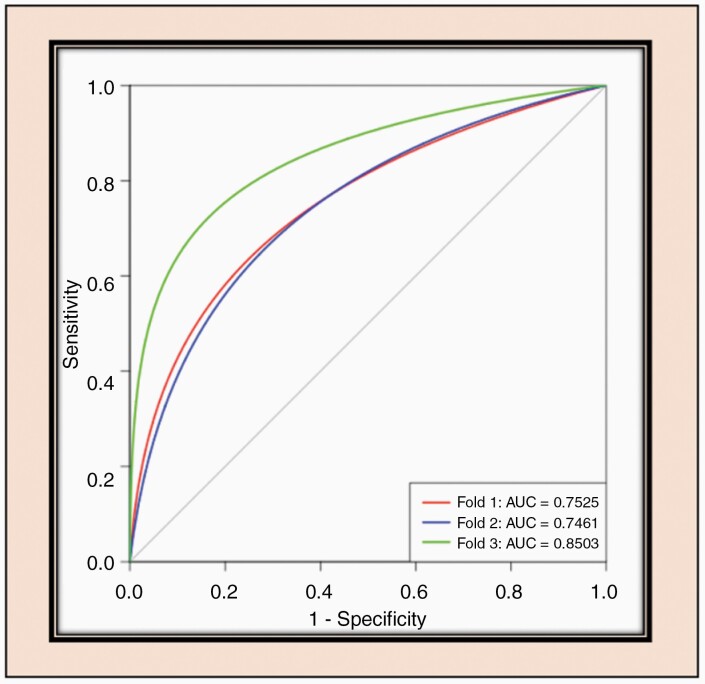
ROC analysis for 1p.19q-net. Separate curves are plotted for each cross-validation fold along with corresponding AUC value.
